# Supplementary material for: SOX4 Mediates ATRA-Induced Differentiation in Neuroblastoma Cells
Source: Cancers (Basel). 2022 Nov 17;14(22):5642. doi: 10.3390/cancers14225642 (PMC9688885; doi:10.3390/cancers14225642)
Supplement: Supplementary file 1 [file cancers-14-05642-s001.zip › Figure S3-S5.pdf]

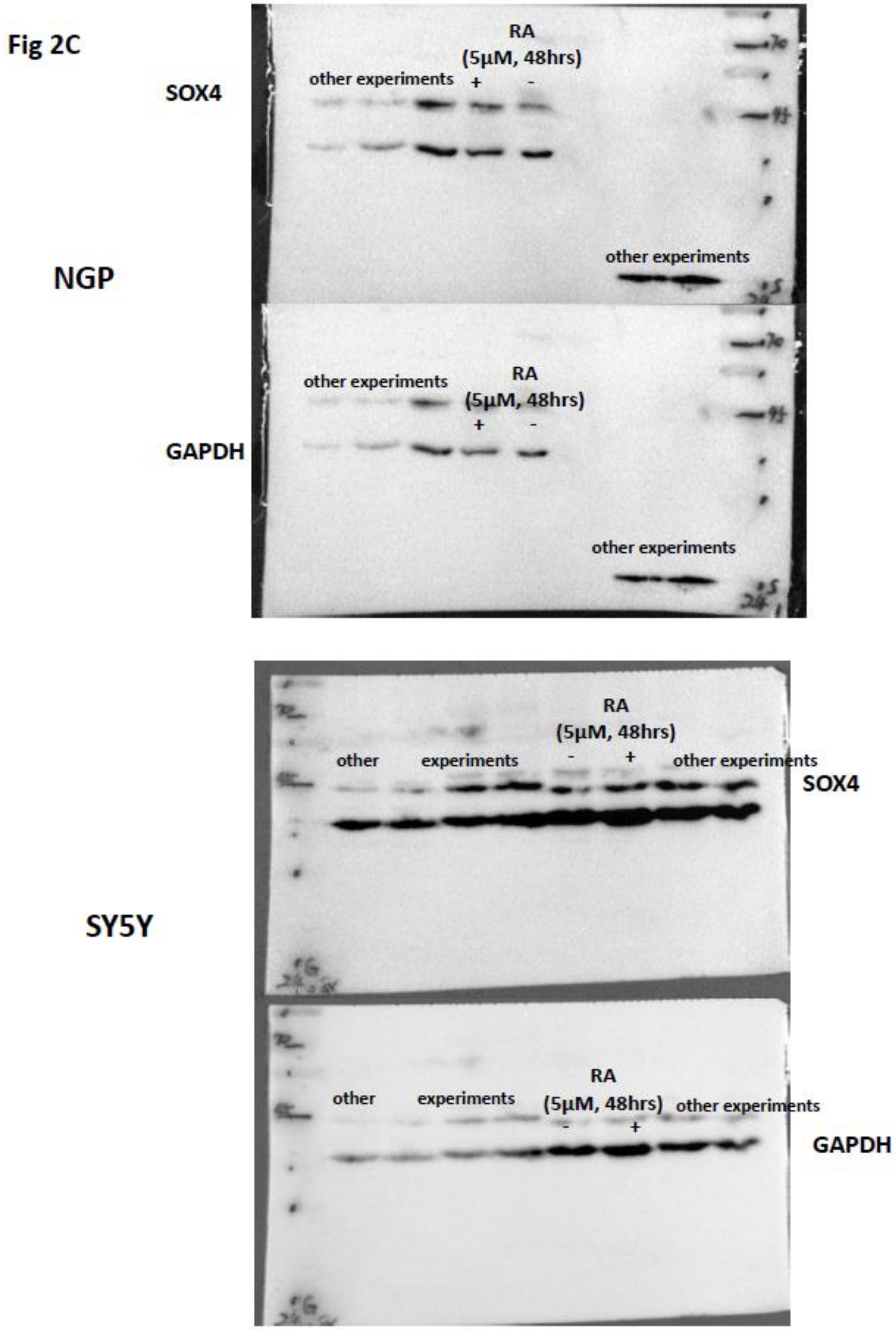

Figure S3. Uncropped western blot of Figure 2C.

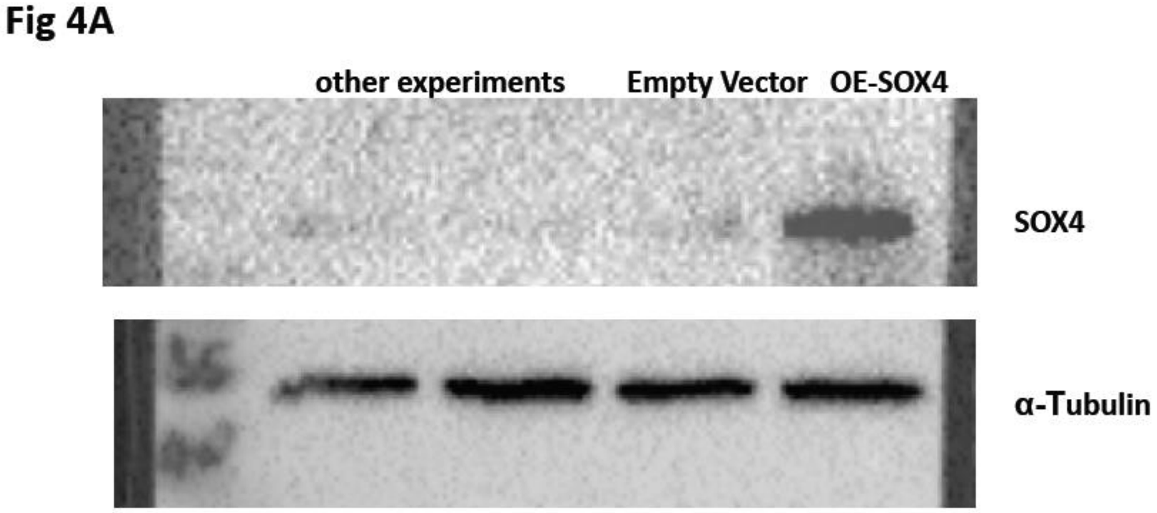

Figure S4. Uncropped western blot of Figure 4A.

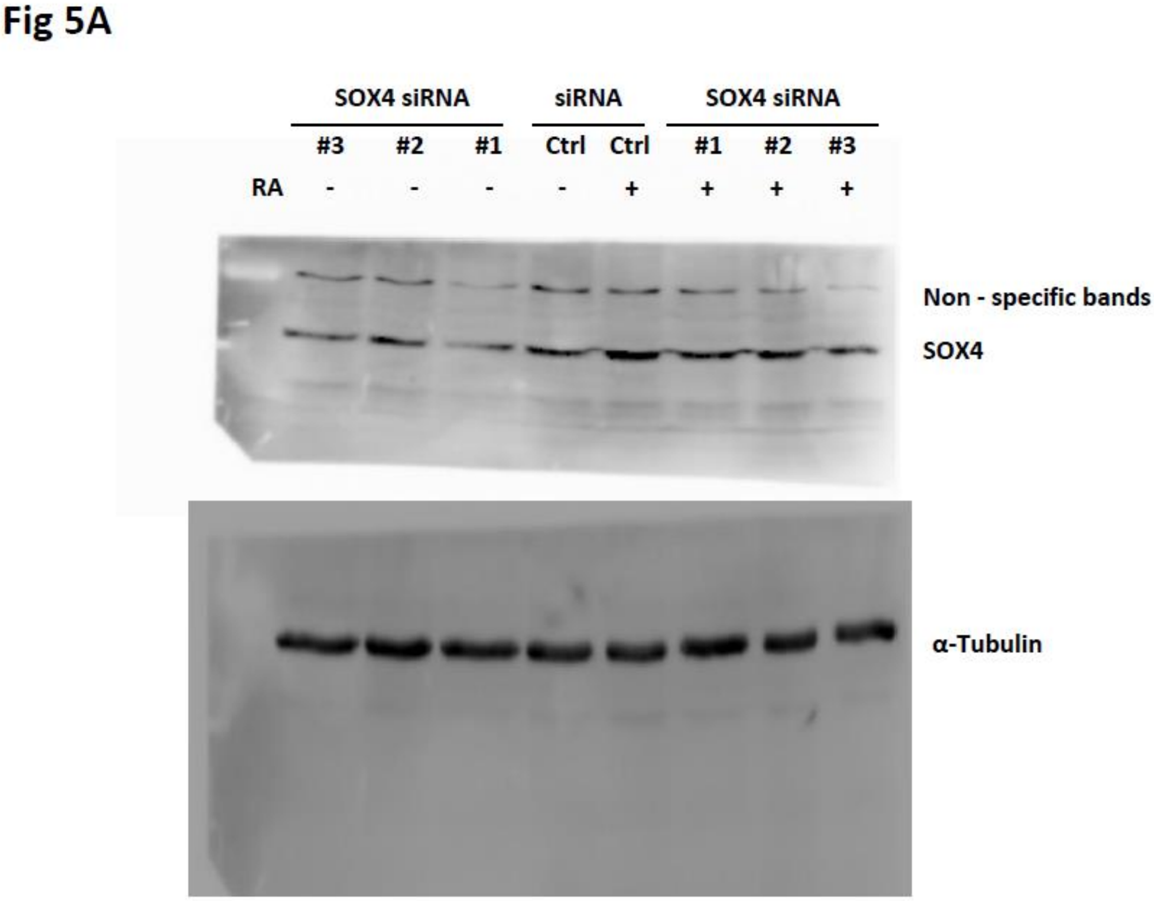

Figure S5. Uncropped western blot of Figure 5A.
