# Supplementary figures and images for: SOX4 Mediates ATRA-Induced Differentiation in Neuroblastoma Cells
Source: Cancers (Basel). 2022 Nov 17;14(22):5642. doi: 10.3390/cancers14225642 (PMC9688885; doi:10.3390/cancers14225642)

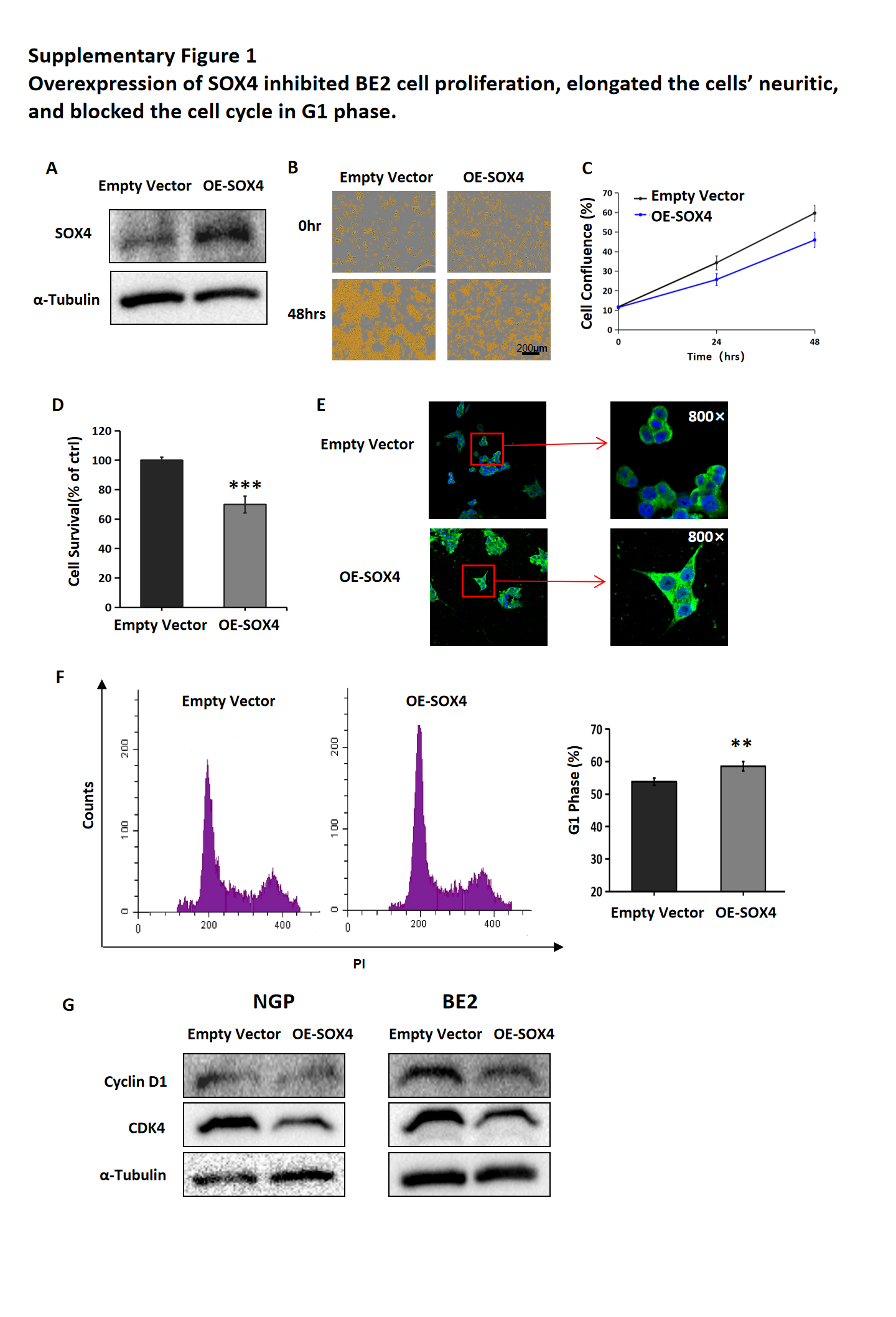

Supplement: Supplementary file 1 [file cancers-14-05642-s001.zip › Figure S1.tif]

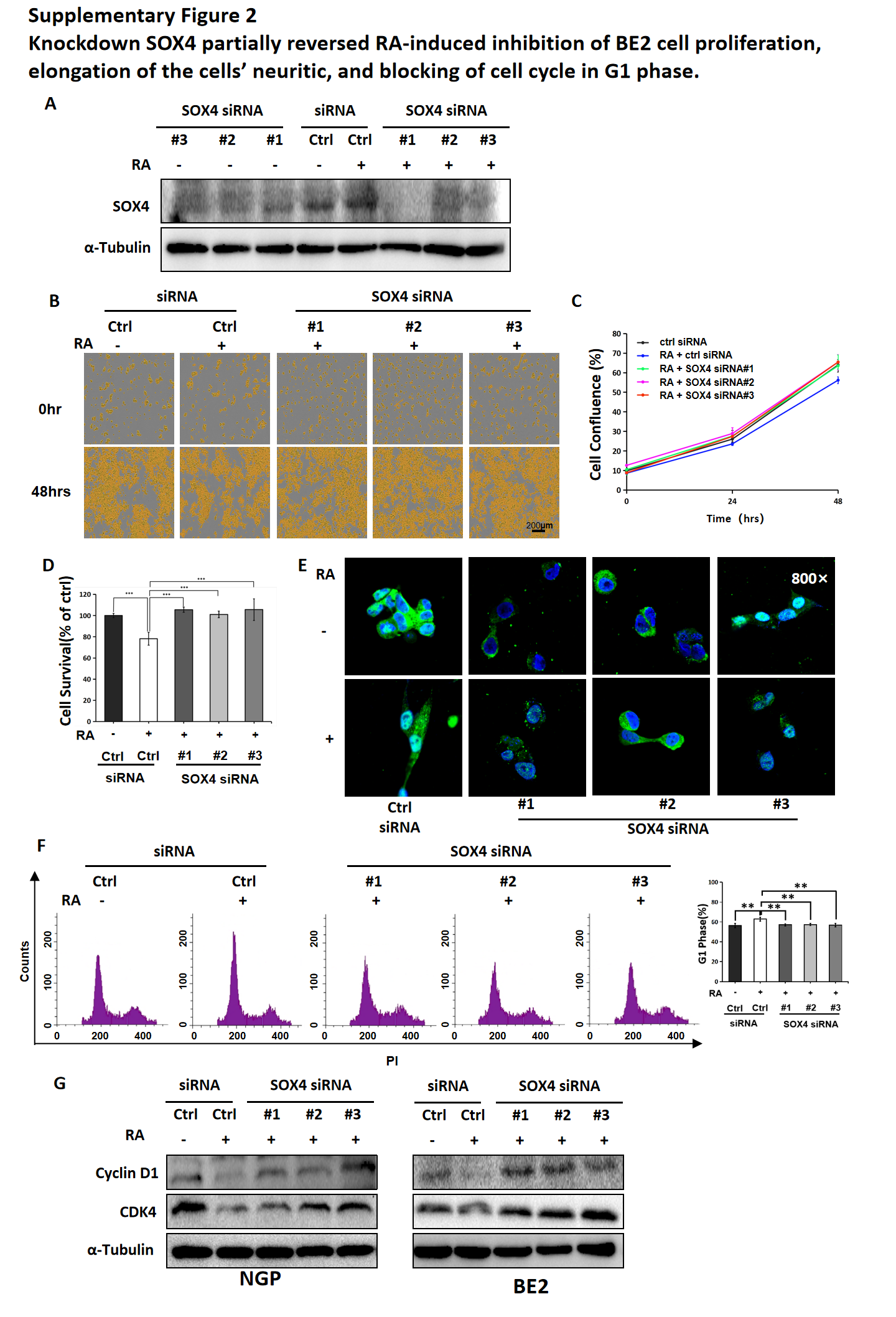

Supplement: Supplementary file 1 [file cancers-14-05642-s001.zip › Figure S2.tif]
